# Valuing Bioactive Lipids from Green, Red and Brown Macroalgae from Aquaculture, to Foster Functionality and Biotechnological Applications

**DOI:** 10.3390/molecules25173883

**Published:** 2020-08-26

**Authors:** Diana Lopes, Tânia Melo, Felisa Rey, Joana Meneses, Fátima Liliana Monteiro, Luisa A. Helguero, Maria Helena Abreu, Ana Isabel Lillebø, Ricardo Calado, Maria Rosário Domingues

**Affiliations:** 1 Mass Spectrometry Centre, LAQV REQUIMTE, Department of Chemistry, University of Aveiro, Santiago University Campus, 3810-193 Aveiro, Portugal; taniamelo@ua.pt (T.M.); felisa.rey@ua.pt (F.R.); joanamf@ua.pt (J.M.); 2 Centre for Environmental and Marine Studies, CESAM, ECOMARE, Department of Biology, University of Aveiro, Santiago University Campus, 3810-193 Aveiro, Portugal; lillebo@ua.pt (A.I.L.); rjcalado@ua.pt (R.C.); 3 Centre for Environmental and Marine Studies, CESAM, Department of Chemistry, University of Aveiro, Santiago University Campus, 3810-193 Aveiro, Portugal; 4 iBIMED-Institute of Biomedicine, Department of Medical Sciences, Universidade de Aveiro, Agra do Crasto, 3810-193 Aveiro, Portugal; lili.flrvm@gmail.com (F.L.M.); luisa.helguero@ua.pt (L.A.H.); 5 ALGAplus-Production and Trading of Seaweeds and Derived Products Lda., 3830-196 Ílhavo, Portugal; helena.abreu@algaplus.pt

**Keywords:** antioxidant, anti-inflammatory, antiproliferative, fatty acids, functional foods, lipids, bioactivities

## Abstract

Marine edible macroalgae have functional proprieties that might improve human health and wellbeing. Lipids represent a minor fraction of macroalgae, yet with major interest as main carriers of omega 3 polyunsaturated fatty acids and intrinsic bioactive properties. In this study, we used lipid extracts from the green macroalgae *Ulva rigida* and *Codium tomentosum*; the red *Gracilaria gracilis,* *Palmaria palmata* and *Porphyra dioica;* and the brown *Fucus vesiculosus*, produced in a land-based integrated multitrophic aquaculture (IMTA) system. We determined the lipid quality indices based on their fatty acid profiles and their bioactivities as putative antioxidant, anti-inflammatory and antiproliferative agents. The results reveal to be species-specific, namely *U. rigida* displayed the lowest atherogenicity and thrombogenicity indices. *Palmaria palmata* and *F. vesiculosus* lipid extracts displayed the lowest inhibitory concentration in the free radical scavenging antioxidant assays. *Ulva rigida, C. tomentosum, P. palmata* and *P. dioica* inhibited COX-2 activity by up to 80%, while *P. dioica* and *P. palmata* extracts showed the highest cytotoxic potential in the MDA-MB-231 breast cancer cells. This work enhances the valorization of macroalgae as functional foods and promising ingredients for sustainable and healthy diets and fosters new applications of high-valued algal biomass, in a species-specific context.

## 1. Introduction

Marine organisms are untapped sources of bioactive molecules with unique properties and multiple potential applications in nutraceutical, cosmeceutical, pharmaceutical and functional food products [1]. Marine macroalgae are popular aquatic resources that have been used for centuries by Asian populations in food, fertilizer and medicinal applications [2]. Macroalgae consumption has been growing in Western countries due to Asian food popularity, globalization and its nutritional attributes and health benefits.

Nowadays, consumers are becoming aware about their diet and the sustainability of consumed products, increasing the demand for healthy and nutritive foods with low ecological impact. Macroalgae are a great example of this, since marine water is not a limited resource as is freshwater [3]. Macroalgae are a source of dietary fibers, proteins, amino acids, vitamins and minerals. They are recognized as functional foods whose consumption, either raw or as an ingredient in different formulations, might provide health benefits such as reducing the risk of chronic diseases, enhancing the ability to manage chronic disorders and improving health state and wellbeing [4]. An inverse relationship between regular macroalgae consumption and reduced risk of hypertension and cardiovascular diseases has been probed [5,6,7]. One example are the epidemiological studies from Japan and South Korea, where populations are great consumers of macroalgae, showing a decrease in obesity and dietary-related diseases, as well as higher longevity [8,9].

The world production of marine macroalgae has more than tripled, up from 10.6 million tons in 2000 to 32.4 million tons in 2018 [10]. Some edible macroalgae species are well-suited for marine aquaculture production [11,12], to be included in healthy and sustainable diets, and labeled as organic products [13]. Novel foods with health benefits are more attractive for consumers, as revealed by a preference study on Italian consumers, where 76% were willing to eat macroalgae [14]. Furthermore, marine aquaculture production minimizes toxicity risks associated with potential high iodine concentrations or accumulation of arsenic in their tissues [4,15] that can occur by the accumulation of these elements from seawater. Marine macroalgae can be classified in three major groups, namely green (Phylum Chlorophyta), red (Phylum Rhodophyta) and brown (Phylum Ochrophyta formerly named Phaeophyta), each characterized by a distinctive combination of photopigments. Overall, their growing interest is powered by their bioactive molecules with several industrial applications [16]. The most well-studied macroalgae bioactive compounds include abundant molecules such as polysaccharides and peptides, although minor constituents, such as alkaloids and lipids, have also been recognized for their nutritional and bioactive values [17,18]. Even though macroalgae have a low lipid content (which is variable among macroalgal species [19,20,21,22,23]), these compounds are recognized as an important source of polyunsaturated fatty acids (PUFA) including long-chain omega-3 FA, such as α-linolenic acid (18:3*n*-3), eicosapentaenoic acid (20:5*n*-3) and docosahexaenoic acid (22:6*n*-3), that have been addressed as essential modulators to reduce the risk of cancer and cardiovascular diseases [24]. Omega-3 PUFA mostly occurs in their esterified form in polar lipids, mainly as glycolipids and phospholipids, which were recently identified in edible green, red and brown macroalgae species *Ulva rigida, Codium tomentosum, Gracilaria* sp.*, Palmaria palmata, Porphyra dioica* and *Fucus vesiculosus* produced under controlled conditions on a sustainable land-based integrated multitrophic aquaculture (IMTA) system [19,20,21,23,25,26]. The polar lipidomic profiling performed in these studies unveiled the presence of potential bioactive lipid species belonging to glycolipid and phospholipid. Screening for lipid extracts bioactivity has been done for few of these edible macroalgae, showing the antioxidant activity of *P. palmata* and *Porphyra* sp. lipid extracts [20,27], the anti-inflammatory activity of *P. palmata* and *Fucus spiralis* polar lipids [28,29] and the antiproliferative action of *Gracilaria* sp. and *Porphyra crispara* polar lipids [25,30].

Macroalgae’s lipids and biomass have been more frequently applied as ingredients for functional food, cosmeceutical, nutraceutical and feed enrichment purposes [31]. The possibility to include macroalgae in meat (e.g., sausages and burgers), plant-based food products (e.g., bread, pasta, etc.) and yogurts to improve their quality, functional properties and increase shelf life has been explored [32,33]. Overall, an in depth look into the bioactive properties of lipids from macroalgae will certainly promote the valorization of these marine biological resources and favor new value-added applications.

Previous studies have shown that macroalgae from different phyla, as well as from different species of the same phylum, display distinct lipid profiles, which can have an impact on the nutritional and functional value of the macroalgae’s biomass [19,20,21,23,26]. Understanding how these differences can be reflected in their bioactive potential is paramount to advance the state of the art of macroalgae’s potential. The present study aimed to evaluate the biological activities and quality indices of the lipid extracts of a set of edible macroalgae cultivated under the same IMTA conditions: the green macroalgae *Ulva rigida* C. Agardh (1823) and *Codium tomentosum* Stackhouse (1797); the red *Gracilaria gracilis* (Stackhouse) Steentoft, L. M. Irvine & Farnham (1995), *Palmaria palmata* (Linnaeus) F. Weber & D. Mohr (1805) and *Porphyra dioica* J. Brodie & L. M. Irvine (1997); and the brown *Fucus vesiculosus* Linnaeus (1753). Lipid extracts of each macroalga were used to characterize their FA profile and evaluate their antioxidant (ABTS and DPPH assays) and anti-inflammatory (COX-2 assay) activity and effect on breast cancer cells proliferation (cell viability assay).

## 2. Results

### 2.1. Fatty Acid Profiles and Lipid Quality Indices

Fatty acid profiles present in the lipid extracts of green *U. rigida* and *C. tomentosum*; red *G. gracilis*, *P. palmata* and *P. dioica*; and brown *F. vesiculosus* are summarized in Table 1. The identified FA corresponded to the FA esterified to glycerolipids and were derivatized by transesterification reactions and analyzed as fatty acid methyl esters (FAME). The palmitic acid (16:0) was identified in all macroalgae with an abundance in the order of 20%, with the exception of *F. vesiculosus* (11.9 ± 0.5%). Some of the FA that were present in higher proportions were 20:4*n*-6 in *G. gracilis* (with a relative abundance of 35.4 ± 1.5%) and eicosapentaenoic acid (20:5*n*-3, EPA) in *P. palmata* (with a relative abundance of 51.9 ± 6.5%). It is also noteworthy the presence of omega-3 FA in *U. rigida*, namely 18:4*n*-3 and α-linolenic acid (18:3*n*-3, ALA) with relative abundance of 24.4 ± 0.4% and 10.9 ± 0.4%, respectively, and also the FA 16:4*n*-3 and 22:5*n*-3 with relative abundance of 19 ± 0.6% and 4.1 ± 0.1%, respectively.

The atherogenicity index (AI) and thrombogenicity index (TI) were calculated from the FA profiles of *U. rigida*, *C. tomentosum*, *Gracilaria* sp., *P. palmata*, *P. dioica* and *F. vesiculosus* and are summarized in Table 2.

The lowest AI were recorded in *U. rigida* (0.3 ± 0.0), *C. tomentosum* (0.6 ± 0.1) and *G. gracilis* (0.6 ± 0.0) with no significant differences between them. The lowest TI were recorded by *U. rigida* (0.1 ± 0.0) *C. tomentosum* (0.2 ± 0.0) and *P. palmata* (0.2 ± 0.1) with no significant differences between them. Overall, the macroalga *U. rigida* achieved the lowest AI and TI indices.

### 2.2. Antioxidant Activity

The antioxidant activity of *U. rigida*, *C. tomentosum*, *P. palmata*, *G. gracilis*, *P. dioica* and *F. vesiculosus* lipid extracts was evaluated using free radical ABTS^●+^ and DPPH^●^ scavenging assays. The lipid extracts of all macroalgae promoted a 50% inhibition (IC50) in the ABTS^●+^ assay, while in DPPH^●^ assay only a 20% inhibition (IC20) was recorded for all lipid extracts. The Trolox equivalent (TE) and concentration of lipid extracts to achieve inhibitions of 50% and 20% varied with macroalgae and are summarized in Table 3.

In ABTS^●+^ assay, the lowest IC50 was recorded for the lipid extract of the macroalga *P. palmata* followed by *F. vesiculosus* and *U. rigida*, with IC50s of 23.7 ± 0.6, 27.3 ± 0.2 and 30.7 ± 0.1 µg/mL and TE of 606.1 ± 14.6, 507.1 ± 3.5 and 500.5 ± 1.7 μmol Trolox/g lipid, respectively. For the macroalgae *P. dioica* and *C. tomentosum*, the IC50s were 41.1 ± 2.5 and 48.1 ± 0.03 µg/mL corresponding to TE of 338.8 ± 20.5 and 327.9 ± 0.2 μmol Trolox/g lipid, respectively. The macroalga *G. gracilis* reached the highest IC50 at 86.4 ± 3.4 µg/mL and TE at 183.0 ± 7.1 μmol Trolox/g lipid. Significant statistical differences in IC50 and TE values were recorded between different algae in ABTS^●+^ assay, as detailed in Table 3.

In DPPH^●^ assays, there were four species of macroalgae reaching the lowest IC20 in the same order of magnitude, *F. vesiculosus* with an IC20 of 106.0 ± 5.6 µg/mL and a TE of 89.7 ± 4.6 μmol Trolox/g lipid, followed by *G. gracilis* (IC20 119.5 ± 1.8 µg/mL; TE 89.2 ± 1.3 μmol Trolox/g lipid), *P. palmata* (IC20 119.6 ± 8.0 µg/mL; TE 89.5 ± 6.3 μmol Trolox/g lipid) and *U. rigida* (IC20 120.8 ± 3.8 µg/mL; TE 88.0 ± 2.8 μmol Trolox/g lipid). The macroalgae *P. dioica* and *C. tomentosum* achieved an IC20 of 212.5 ± 7.0 and 249.9 ± 66.7 µg/mL and a TE of 44.9 ± 1.5 and 47.5 ± 12.7 μmol Trolox/g lipid, respectively. Significant differences between macroalgae were recorded in IC20 in DPPH^●^ assay but not in its TE (Table 3).

### 2.3. Anti-Inflammatory Activity

**Figure 1:**
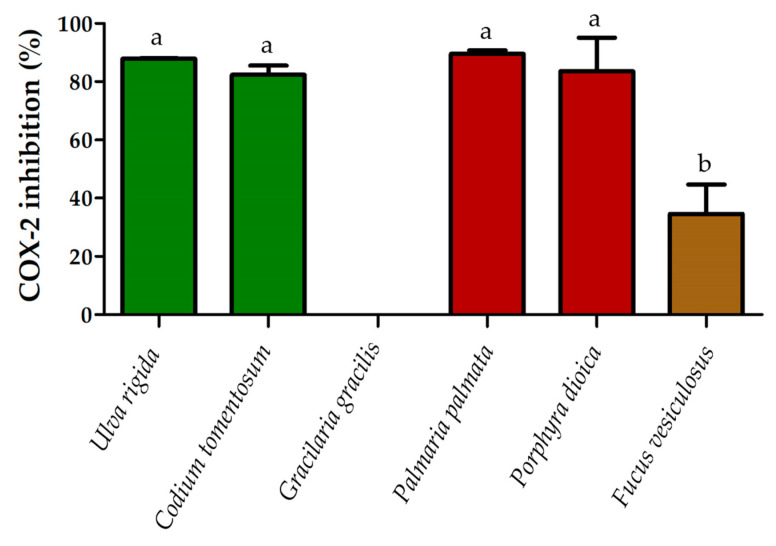
Inhibition of COX-2 activity (expressed in percentage, %) demonstrated by the lipid extracts of *Ulva rigida*, *Codium tomentosum*, *Palmaria palmata*, *Porphyra dioica* and *Fucus vesiculosus* at a concentration of 500 µg/mL. Values are averages of three assays (*n* = 3) ± standard deviation. In the case of *Gracilaria gracilis*, no inhibition was obtained. Different letters indicate significant differences between species (*p* < 0.05, ANOVA followed by Tukey’s HSD post hoc analysis).

The anti-inflammatory activity of *U. rigida*, *C. tomentosum*, *G. gracilis*, *P. palmara*, *P. dioica* and *F. vesiculosus* lipid extracts was determined through cyclooxygenase (COX-2) activity assay (Figure 1).

The highest percent of inhibition of COX-2 activity was recorded for the lipid extracts of *P. palmata* with 89.5 ± 0.9%, followed by *U. rigida* that promoted a COX-2 inhibition of 87.9 ± 0.1%. The lipid extracts of *P. dioica* and *C. tomentosum* induced 83.6 ± 8.1% and 82.3 ± 2.2% of inhibition, respectively. Only the lipid extracts of two macroalgae achieved less than 50% of inhibition, those of *F. vesiculosus* with 34.6 ± 7.1% and *G. gracilis* that showed no inhibition potential of COX-2 in this assay.

### 2.4. Effect on Breast Cancer Cell Proliferation

**Figure 2:**
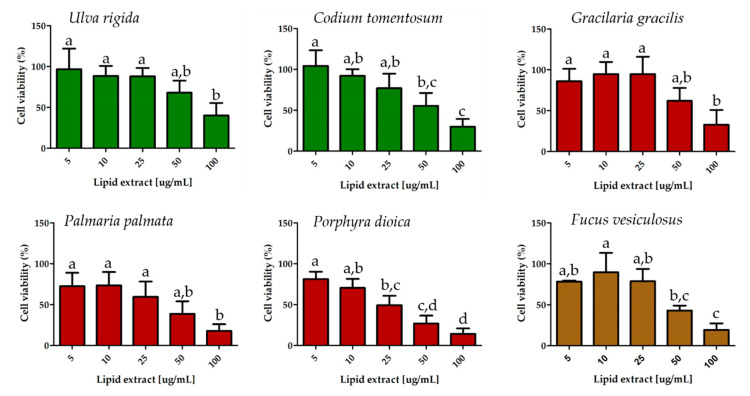
Antiproliferative effect in MDA-MB-231 cell viability of the lipid extracts of *Ulva rigida*, *Codium tomentosum*, *Gracilaria gracilis*, *Palmaria palmata*, *Porphyra dioica* and *Fucus vesiculosus* at five distinct concentrations. Control values obtained for cell culture without lipid extract correspond to cell viability of 100% and are not represented in the bar graphs. Values are presented as the average of three assays (*n* = 3) ± standard deviation (Tukey’s HSD post hoc analysis, *p* < 0.05). Different letters in the same graph represent significant differences among concentrations.

The antiproliferative activity of *U. rigida*, *C. tomentosum*, *G. gracilis*, *P. palmata*, *P. dioica* and *F. vesiculosus* lipid extracts was evaluated measuring the cell proliferation of a highly aggressive MDA-MB-231 breast cancer cell line (Figure 2).

ANOVA test revealed significant differences in MDA-MB-231 cell viability between lipid extract concentrations for all macroalgae: *U. rigida* (*p* = 0.011), *C. tomentosum* (*p* < 0.001), *G. gracilis* (*p* < 0.01), *P. palmata* (*p* < 0.01), *P. dioica* (*p* < 0.001) and F*. vesiculosus* (*p* < 0.001). The antiproliferative effects of the different lipid extract concentrations from the same alga were compared using a post hoc analysis (Tukey’s HSD). The results are present in Figure 2. The lipid extract concentration of each macroalga needed to inhibit 50% of cell proliferation (IC50) was calculated (Table 4).

The macroalgae *P. dioica* and *P. palmata* showed the lowest IC50s at 35.5 ± 10.5 and 40.4 ± 19.2 μg/mL, respectively, followed by the macroalgae *F. vesiculosus* with 52.5 ± 10.9 μg/mL and *C. tomentosum* with 66.4 ± 12 μg/mL. The two macroalgae that required higher concentrations of lipid extract to inhibit 50% of cancer cells growth were *G. gracilis* and *U. rigida*, with IC50s of 74.7 ± 19.1 and 82.7 ± 19.1 μg/mL, respectively (Table 4). There were no significant differences in IC50 between the macroalgae species being compared.

## 3. Discussion

The global increase in consumption of seafood products including macroalgae arises from consumers awareness that these products are an important source of nutrients including proteins, polysaccharides, vitamins and lipids [34,35,36,37]. In macroalgae, lipids are primary metabolites and accounted for approximately 0.2–8% dry weight (DW), while carbohydrates accounted for 12–71% DW and proteins for approximates 4.3–47% DW [19,38]. However, despite the lower abundance of lipids compared with the other primary metabolites, lipids from macroalgae are a natural source of PUFA, particularly omega-3 FA, being an interesting alternative to fish, especially macroalgae produced under sustainable aquaculture practices, because of its cultivation under controlled conditions [23,39]. In addition, the polar lipids of macroalgae have been reported to display several bioactivities [25,40,41], although this research field remains rather untapped [42].

The set of macroalgae covered in this study, originating from three different phyla (Chlorophyta, Rhodophyta and Ochrophyta) showed a FA profile consistent with previous studies [20,21,23,25,26,41]. In fact, FA are usually characteristic of each macroalga species, however the abundance of several FA species can change in relative amounts depending on season and/or growth conditions [19,23]. Although some of the studied species belong to the same phylum, there are clear species-specific FA profiles (Table 1). Some macroalgae extracts have high PUFA content. Higher abundance of EPA was found in *P. palmata* (51.9 ± 6.5%) and *P. dioica* (20.5 ± 2.3%) lipid extracts, and greater abundance of ALA was found in *C. tomentosum* (14 ± 0.6%) and *U. rigida* (10.9 ± 0.4%). This illustrates how variable the nutritional value of macroalgae lipids can be, along with their functional properties.

The composition of FA on a diet has implications in risk factor of coronary artery diseases. There are two measures to evaluate the quality and health benefits of ingested lipids, namely atherogenicity index (AI) and thrombogenicity index (TI) from FA profile, and both are used as cardiovascular risk predictors. Atherogenicity index measures the portion of pro-atherogenic and anti-atherogenic FA, evaluating the ratio between FA that favor the adhesion of lipids to cells of the immunological and circulatory systems, and FA that inhibit the formation of lipid plaques [43,44,45]. On the other hand, TI defines the relationship between the pro-thrombogenic (saturated) and the anti-thrombogenic FA showing the tendency of clots formation in blood vessels [43,44,45]. Higher values of AI and TI (>1.0) are detrimental to human health [46,47]. Macroalgae species studied herein had AI and TI less than or equal to 1.1 (Table 2). Among the set of studied macroalgae, *U. rigida* displayed the highest content of unsaturated FA compared to saturated FA and also the higher proportion of omega-3 FA compared to omega-6 FA, thus supporting the lowest AI and TI. The values reported for these indices are in line with those retrieved from previous studies addressing macroalgae and marine fish, as well as other added value species (e.g., the ragworm *Hediste diversicolor*, used as feed for marine fish and shrimp broodstock) [48,49].

The growing demand for natural compounds with antioxidant activity is transversal to food, pharmaceutical and cosmetical industries [50,51,52]. Antioxidant activities from natural sources are usually associated with their profile and content in phenolic compounds [52]. While phenolic compounds are not commonly abundant in macroalgae, because they are secondary metabolites produced under stress conditions, other compounds such as lipids, particularly PUFA, have already been reported as potent antioxidants but have been less thoroughly studied [53]. Antioxidant activity usualy evaluates the capacity of redox molecules in foods and biological systems to scavenge free radicals [54,55]. In ABTS^●^^+^ assay, all lipid extracts evaluated showed an IC50 lower than 100 μg/mL. Lower IC50 indicates higher radical scavenging activity and antioxidant potential. The lipid extract with the highest IC50 was that from *G. gracilis* (86.4 ± 3.4 μg/mL). The results gathered for the red macroalga *G. gracilis* show a better antioxidant performance in the ABTS*^●^^+^* assay than the ethanolic (0.689 ± 0.045 mg/mL) and methanolic extracts (1.090 ± 0.073 mg/mL) of *Gracilaria manilaensis,* which exhibited high phenolic and flavonoid content [56]. The results of the DPPH*^●^* assay is not comparable with other studies because studied lipid extract only achieved 20% of inhibition and we cannot extrapolate for IC50 values. The oxidative stress is multifactorial, involving species with different reactivity and solubility. Therefore, it is crucial to evaluate antioxidant properties by using different assays with distinct mechanisms to quench, scavenge and reduce both aqueous and lipophilic radicals. This approach will allow gaining a more in-depth knowledge on antioxidant compounds present in the samples being screened. Lipid extracts from the red *P. palmata* and the brown macroalgae *F. vesiculosus* achieved the lowest IC50 and IC20, respectively. Extracts from both species displayed a high content in EPA, which may be related to their potent antioxidant action [53].

The search of compounds with anti-inflammatory activity is yet another area of research which deserves further exploration, as inflammatory diseases have emerged as a prominent health issue [57,58]. Diet plays a relevant role in modulating inflammatory state, highlighting food as a potential therapeutic tool in disorders with an inflammatory basis [57]. Fatty acids composition of diets can control inflammation through biosynthesis of lipid mediators, which derive primarily from membrane phospholipids and reflect dietary FA intake [59]. The ratio between omega-6 and omega-3 PUFA is recognized to play a key role in inflammation [60,61]. While several diseases, such as atherosclerosis, sepsis, mastitis and cancer, involve derivatives of omega-6 PUFA, an increased consumption of omega-3 from marine source was shown to lower the risks of cardiovascular diseases and cancer [62,63,64,65,66,67]. Therefore, the use of lipid extracts rich in omega-3 PUFA from macroalgae may hold great potential to be used as ingredients in the formulation of new products for food and feed enrichment, supplements and nutraceuticals.

In the anti-inflammatory process, the cyclooxygenase (COX) enzyme, which includes the constructive (COX-1) and inducible (COX-2) isoforms, are involved in the synthesis of prostaglandins, thromboxanes and prostacyclins [68,69,70]. The COX-2 isoform is selectively induced by proinflammatory cytokines during inflammation. This enzyme catalyzes prostaglandin biosynthesis from arachidonic acid (20:4*n*-6, AA), and its inhibition translates in subsequent inhibition of prostaglandins, instigating an anti-inflammatory effect [70]. The lipid extracts of *U. rigida*, *C. tomentosum*, *P. palmata* and *P. dioica* achieved over 80% COX-2 inhibition without significant differences being recorded between them. The macroalga *F. vesiculosus* reached lower COX-2 inhibition and the lipid extract of *G. gracilis* did not show any activity. The content of AA, which is the FA with the greatest relative abundance in *G. gracilis* and the second most abundant in *F. vesiculosus*, seems to be the best explanation for the poor results obtained in the lipid extracts of these two macroalgae. The COX-2 enzyme catalyzes the conversion of AA into endoperoxides, which initiates the biosynthetic pathway to the prostaglandins [71]. As the lipid extracts of *G. gracilis* and *F. vesiculosus* contain higher AA content, and considering that the AA is the substrate of COX-2 enzyme, the poorer performance of the lipid extracts from these two macroalgae could somehow be anticipated. To our knowledge there are few publications that evaluated COX-2 inhibition by macroalgae lipid extracts, and the ones published used cell lines assays, but not in vitro COX-2 assays. Previous studies evaluated the COX-2 expression, evidencing a reduction of the enzyme expression in cell lines [72,73,74,75], but polar lipid extracts from terrestrial plants and microalgae showed modulation capacity of COX-2 using *in vitro* assays [75,76]. The ethanolic extract of the small herbaceous plant *Polygonum minus* (Huds) achieved 25% of COX-2 inhibition at concentration of 100 μg/mL [76] which is in the same order of magnitude we obtained in the present study (about 80% inhibition at a concentration of 500 μg/mL). In addition, the ethanolic extract of the microalga *Skeletonema* sp. [77] achieved an inhibition of 82 ± 2% of COX-2 at concentrations of 1 mg/mL, which highlights the potential of the results for the lipid extracts of the macroalgae being reported in this is study.

Some metabolites from macroalgae such as lipids, sterols, terpenoids, pigments and phenolic compounds were tested as chemopreventive and anti-cancer agents [78,79]. The evaluation of antiproliferative activity of lipid extracts from *U. rigida, C. tomentosum, G. gracilis, P. palmata, P. dioica* and *F. vesiculosus* showed that all of these macroalgae have an inhibitory capacity in the range of 10–100 μg/mL. The lowest IC50 was obtained by *P. dioica* and *P. palmata* lipid extracts. A similar study using a lipid extract of *Gracilaria* sp. [25] reported an IC50 of 12.2 μg/mL and 12.9 μg/mL for T-47D and 5637 cancer cells, respectively. Another study that used methanolic extract of *P. palmata* [80], rich in polyphenolic compounds, achieved an IC50 of 2.30 mg/mL in HeLa cell line, which is higher than the concentration reported in our study. The lipid extracts of *P. dioica* and *P. palmata* that showed higher inhibition are those that have a higher content of EPA. It was previously demonstrated that EPA and docosahexaenoic acid (22:6*n*-3, DHA) decrease the proliferation and increase the apoptosis of human breast cancer cells by inhibition of signaling through the Akt/NFκB cell survival pathway [81]. Since Akt requires translocation to the plasma membrane for activation, MDA-MB-231 cell membrane enrichment of omega-3 FA after exposure to omega-3 PUFA might affect the activation of signaling molecules such as Akt that are recruited to the membrane for activation [81]. In addition, omega-3 PUFA reduced DNA binding activity of both p50- and p65-containing NFκB as a result of decreased Akt phosphorylation [81,82,83,84].

The bioactivities of lipid extracts herein demonstrated are not exclusive of FA. The FA are esterified in polar lipids, which have been associated with intrinsic bioactive proprieties, as mentioned for the inhibition of DNA polymerase α/β by sulfolipids and galactolipids as antitumoral agents [85,86,87,88]. The sulfonic acid linkage and the number of ethylenic double bonds in unsaturated FA was reported to be relevant structural features in their action [85,86,87]. Likewise, other polar lipids such as phospholipids from marine origin have anti-inflammatory properties, since phospholipids are better delivers of PUFA than triglycerides [89,90,91].

Several studies have shown that macroalgal lipid profiles are highly prone to change under contrasting biotic and abiotic conditions [23,39,92]. The set of macroalgae present in this study were produced under the same IMTA system conditions. The production of macroalgae in this kind of system allows obtaining more biochemically stable, safer and more sustainable biomass. Moreover, this culture framework makes it possible to fine-tune abiotic conditions that may favor certain lipid profiles, thus allowing to customize the lipid extracts of farmed macroalgae for distinct applications.

The present study unveils the functionality of macroalgal lipid extracts towards diverse species-specific bioactivities, in line with the unique lipid signature of the macroalgae addressed (green *U. rigida* and *C. tomentosum*; red *G. gracilis*, *P. palmata* and *P. dioica*; and brown *F. vesiculosus*) [20,21,22,23,25,26,41]. Even though the experimental protocols employed were specific for lipid extraction, one cannot ignore the putative role that the residual presence of other molecules, such as pigments and phenolic compounds, may have played in our findings. Potential synergistic effect between these residual molecules and lipids should also be investigated in future studies aiming to add value to sustainably farmed macroalgae biomass.

## 4. Materials and Methods

### 4.1. Macroalgae Biomass

Samples of fresh biomass of *U. rigida*, *C. tomentosum*, *G. gracilis*, *P. dioica*, *P. palmata* and *F. vesiculosus* were provided by a local aquaculture producer—ALGAplus (Aveiro, Portugal, 40°36′43″ N, 8°40′43″ W). Macroalgae were produced in an open-flow outdoor tank system integrated with finfish production on earthen ponds. All macroalgae were harvested in May 2018, cleaned to remove the epiphytes and washed using sterilized seawater. Macroalgae were then dried at 25 °C in an air tunnel until 10–12% total moisture was reached. Five (*n* = 5) 250 mg aliquots were obtained from bulk production of each macroalga species and were used for total lipid extraction.

### 4.2. Lipid Extraction

Macroalgal biomass was grounded using a mortar and pestle with liquid nitrogen. Lipid extracts were prepared according to a modified Bligh and Dyer protocol [25]. Briefly, 250 mg of macroalgae biomass (five replicates) were mixed with 2.5 mL of MeOH and 1.25 mL of CH_2_Cl_2_ in a glass PYREX tube and homogenized by vortexing for 2 min, followed by an incubation period of 2 h and 30 min on ice on a rocking platform shaker (Stuart equipment, Bibby Scientific, Stone, UK). The mixture was centrifuged (Selecta JP Mixtasel, Abrera, Barcelona, Spain) for 10 min at 2000 rpm and the organic phase collected. The biomass residue was re-extracted twice with 2.5 mL of MeOH and 1.25 mL of CH_2_Cl_2_. To wash the lipid extract and induce phase separation, 2.25 mL of Milli-Q water and 1.25 mL of CH_2_Cl_2_ were added to the final organic phase, followed by a new centrifugation for 10 min at 2000 rpm. The organic lower phase was collected and transferred to amber vials that were dried under nitrogen stream, weighed and stored at −20 °C. Lipid content was estimated as a dry weight percentage.

### 4.3. Fatty Acid Analysis

Gas chromatography mass spectrometry (GC-MS) was used to determine FA profile of each macroalgae species. FA methyl esters (FAMEs) were prepared using a methanolic solution of potassium hydroxide (2.0 M) [93]. A sample volume of 2 μL of hexane solution containing FAMEs was analyzed by GC-MS on a GC system (Agilent Technologies 6890 N Network, Santa Clara, CA, USA) equipped with a DB-FFAP column with the following specifications: 30 m long, 0.32 mm internal diameter and 0.25 μm film thickness (123–3232, J & W Scientific, Folsom, CA, USA). The GC equipment was connected to an Agilent 5973 Network Mass Selective Detector operating with an electron impact mode at 70 eV and scanning range *m/z* of 50 to 550 in a one second cycle in full scan mode acquisition. The oven temperature was programmed from an initial temperature of 80 °C for 3 min, a linear increase to 160 °C at 25 °C/min, followed by linear increase at 2 °C min^−1^ to 210 °C, then at 30 °C min^−1^ to 250 °C, and standing at 250 °C for 10 min. The injector and detector temperatures were 220 and 280 °C, respectively. Helium was used as the carrier gas at a flow rate of 1.4 mL/min. FA identification was performed considering the retention times and MS spectra of FA standards (Supelco 37 Component Fame Mix, Sigma-Aldrich, St. Louis, MO, USA), and by MS spectrum comparison with chemical databases (Wiley 275 library and AOCS lipid library). FAMEs of five analytical replicates were injected. The relative amounts of FAs were calculated by the percent relative area method with proper normalization using internal standard methyl nonadecanoate (C19:0, Sigma-Aldrich), considering the sum of all relative areas of the identified FA. Atherogenicity (AI) and thrombogenicity (TI) indices were calculated as follows:

(AI) = [C12:0 + (4 × C14:0) + C16:0]/(omega-3PUFA + omega-6PUFA + MUFA)(1)

(TI) = [C14:0 + C16:0 + C18:0]/[(0.5 × C18:1) + (0.5 × sum of other MUFA) + (0.5 × omega-6PUFA) + (3 × omega-3PUFA) + omega-3PUFA/omega-6PUFA)](2)

### 4.4. 2,2′-Azino-bis-3-Ethylbenzothiazoline-6-Sulfonic Acid Radical Cation Assay—ABTS Radical Scavenging Activity

The antioxidant scavenging activity against the 2,2′-azino-bis-3-ethylbenzothiazoline-6-sulfonic acid radical cation (ABTS^●+^) was evaluated using a previously described method [94,95] with some modifications. The ABTS radical solution (3.5 mmol/L) was prepared by mixing 10 mL of ABTS stock solution (7 mmol/L in water) with 10 mL of potassium persulfate, K2S2O8 (2.45 mmol/L in water). This mixture was kept for 12 h at room temperature and was diluted in ethanol to obtain an absorbance value of ~0.9 measured at 734 nm using a UV–vis spectrophotometer (Multiskan GO 1.00.38, Thermo Scientific, Hudson, NH, USA) controlled by the SkanIT software version 3.2 (Thermo Scientific). For an evaluation of the radical stability, a volume of 150 μL of ethanol was added to 15 wells microplate followed by addition of 150 μL of ABTS^●+^ diluted solution and an incubation period of 120 min, with absorbance measured at 734 nm every 5 min. For the evaluation of the radical scavenging potential, a volume of 150 μL of each lipid extract from each macroalgae species (12.5 (only for *P. palmata*), 25, 50, 100 and 250 μg/mL in ethanol) or 150 μL of Trolox standard solution (4, 8, 16, 28, 40 and 56 μmol/L in ethanol) were placed in each well followed by addition of 150 μL of ABTS^●+^ diluted solution, and a new incubation period of 120 min, with absorbance measurements at 734 nm every 5 min. The control lipid extracts were also assayed by replacing 150 μL of ABTS^●+^ diluted solution by 150 μL of ethanol. Radical reduction by antioxidant compounds present in the lipid extracts was monitored by measuring the decrease in absorbance during the reaction, thereby quantifying radical scavenging activity, which is accompanied by a radical color change. All measurements were performed in triplicate. The percent of ABTS^●+^ remaining after reaction with antioxidant compounds and percent of inhibition was calculated as follows:

% ABTS^●+^ remaining = (Abs samples after 120 min/Abs sample at the beginning of reaction) × 100 (3)

Inhibition% = ((Abs ABTS^●+^ − (Abs samples − Abs control))/Abs ABTS^●+^) × 100 (4)

The concentration of samples able to reduce 50% of ABTS radical after 120 min (IC50) was calculated by linear regression using the concentration of samples and the percentage of the inhibition curve. The activity is expressed as TE (μmol Trolox/g of sample), according to:

TE = IC50 Trolox (μmol/L) × 1000/IC50 of samples (μg/mL)(5)

### 4.5. 2,2-Diphenyl-1-Picrylhydrazyl Radical Assay—DPPH Radical Scavenging Activity

The antioxidant scavenging activity against the 2,2-diphenyl-1-picrylhydrazy radical (DPPH^●^) was evaluated using a previously described method [94,95] with some modifications. A stock solution of DPPH^●^ in ethanol (250 μmol/L) was prepared and diluted to provide a working solution with an absorbance value of ~0.9 measured at 517 nm using a UV–vis spectrophotometer (Multiskan GO 1.00.38, Thermo Scientific, Hudson, NH, USA) controlled by the SkanIT software version 3.2 (Thermo Scientific). To evaluate the radical stability, a volume of 150 μL of ethanol was added to 15 microplate wells followed by addition of 150 μL of DPPH^●^ diluted solution and an incubation period of 120 min, with absorbance measurement at 517 nm every 5 min. For evaluation of the radical scavenging potential, a volume of 150 μL of each lipid extract from each macroalgae species (25, 50, 100, 250 μg/mL in ethanol) or 150 μL of Trolox standard solution (5, 12.5, 25, 37.5 μmol/L in ethanol) were placed in each well followed by addition of 150 μL of DPPH^●^ diluted solution, and again an incubation period of 120 min, with absorbance measurement at 517 nm every 5 min. Control lipid extracts were also assayed by replacing 150 μL of DPPH^●^ diluted solution by 150 μL of ethanol. Radical reduction by antioxidant compounds present in the lipid extracts was monitored by measuring the decrease in absorbance during the reaction, thus quantifying radical scavenging activity, which is accompanied by a radical color change. All measurements were performed in triplicate. The percent of DPPH radical remaining after reaction with antioxidant compounds and percent of inhibition was calculated as followed:

% DPPH^●^ remaining = (Abs samples after 120 min/Abs sample at the beginning of reaction) × 100 (6)

Inhibition% = ((Abs DPPH^●^ − (Abs samples − Abs control))/Abs DPPH^●^) × 100 (7)

The concentration of samples capable of reducing 20% of DPPH radical after 120 min (IC20) was calculated by linear regression using the concentration of samples and the percentage of the inhibition curve. The activity is expressed as TE (μmol Trolox/g of sample), according to:

TE = IC20 Trolox (μmol/L) × 1000/IC20 of samples (μg/mL)(8)

### 4.6. In Vitro Cyclooxygenase Inhibition Assay

Lipid extracts were dissolved in 100% DMSO at 500 μg/mL. The inhibition potential against COX-2 was carried out by enzyme immunoassay (EIA) using in vitro enzymatic assay kit (catalog No. 701230, Cayman Chemical Company, Ann Arbor, MI, USA), carried out according to the instructions provided by the manufacturer (https://www.caymanchem.com/pdfs/701230.pdf). The COX-2 inhibitor screening assay measures the amount of prostaglandin F_2α_ generated from AA in the cyclooxygenase reaction, which was determined by spectrophotometry at 412 nm using a Multiskan GO 1.00.38 (Thermo Scientific, Hudson, NH, USA) controlled by the SkanIT software version 3.2 (Thermo Scientific). The results were expressed as percentage of inhibited COX-2.

### 4.7. Cell Viability Assay on MDA-MB-231 Breast Cell Line

The antiproliferative activity of macroalgal lipid extracts was evaluated in MDA-MB-231 human breast cancer cell line using crystal violet colorimetric assay to determine the viability of cultured cells. Tumor cells were cultivated in Dulbecco’s Modified Eagle Medium (DMEM-F12, Invitrogen Life Technologies, Paisley, UK) with 10% fetal bovine serum (FBS; Gold, PAA) and 50 μg/mL penicillin/streptomycin (PEST) (Invitrogen) in a humidified incubator at 37 °C under an atmosphere of 5% CO_2_. Ten thousand cells per well were plated on 96-well plates and allowed to attach for 24 h; 100 µL of cell suspension (1–2 × 10^4^ cell/well in complete medium) were used. Following this step, 200 µL of the treatment solution in a range of 5–100 μg/mL were applied to the culture. The lipid extract was dissolved in ethanol and diluted to a final concentration of 0.1% ethanol in phenol-red free DMEM-F12 medium supplemented with 2% charcoal treated FBS (DCC), 2 mM glutamine and 50 μg/mL PEST. The same concentration of ethanol was used in untreated controls. The treatment medium was changed after 48 h incubation and cells allowed to grow for additional 24 h. At the end of incubation, the growth medium was removed, cells were fixed with 4% paraformaldehyde for 20 min and stained with 0.5% crystal violet for 20 min [96]. Thereafter, wells were washed and air dried for 24 h, and then methanol was added to solubilize the pigment and absorbance was measured at 570 nm. Experiments were carried out in quadruplicates and three independent experiments were performed.

### 4.8. Statistical Analysis

Kruskal–Wallis tests followed by Dunn’s post-hoc comparisons were performed to identify significant differences between macroalga species in AI, TI, IC50 and TE from ABTS^●+^ antioxidant assay, IC20 and TE from DPPH^●^ antioxidant assay and IC50 of MDA-MB-231 cells assay. *p*-values were corrected for multiple testing using Benjamin-Hochberg method (*q* values). Differences with *q*-value < 0.05 were considered statistically significant. Differences in the percentage of COX-2 inhibition between different macroalgal lipid extracts, the antiproliferative activity (MDA-MB-231 cell viability) of lipid extracts at different concentrations and IC50 of MDA-MB-231 cells from different macroalgal lipid extracts were analyzed using one-way ANOVA. Post hoc Tukey’s HSD test was used to investigate significant differences between lipid extracts concentrations from the same algae in the antiproliferative activity test. Assumptions of normality and homogeneity of variance were verified prior to analysis through Shapiro–Wilks and Levene’s tests, respectively. Statistical analyses were performed using R 3.6.0 [97] in RStudio version 1.1.442 [98]. Experimental data from FA analysis are shown as a mean ± standard deviation of 5 replicates per macroalga species (*n* = 5), while, for bioactivities, data are shown as a mean ± standard deviation of 3 replicates (*n* = 3).

## 5. Conclusions

This study demonstrates the functionality of lipid extracts from macroalgae sustainably produced in aquaculture. The set of green (*U. rigida* and *C. tomentosum*), red (*G. gracilis*, *P. dioica* and *P. palmata*) and brown (*F. vesiculosus*) macroalgae distributed in three phyla (Chlorophyta, Rhodophyta and Ochrophyta) showed species-specific results for the different bioactivity tests. The lipid quality indices of *U. rigida* achieved the lower AI and TI. For antioxidant potential (ABTS*^●+^* and DPPH^●^ assays), the lower IC and higher TE were obtained for *P. palmata* and *F. vesiculosus*, respectively. The macroalgae *U. rigida*, *C. tomentosum*, *P. palmata* and *P. dioica* showed the most potent anti-inflammatory potential. The macroalgae *F. vesiculosus* and *G. gracilis* showed low and no inhibition, respectively, possibly due to their content in AA, which represents an assay limitation. The antiproliferative activity in breast cancer cells reached the lowest IC50 for the red *P. dioica* and *P. palmata* lipid extracts. The lipid content already reported in previous studies along with their PUFA compositions is likely the main cause for the different bioactivities; however, the presence of other lipid-soluble molecules and the synergistic effect between them should not be ignored. This work contributes to the species-specific valorization of macroalgae in line with ONU 2030 sustainable development goals (SDG). Its contribution fulfills the gap on the need of sustainable health diets and new therapeutic strategies for disease prevention of non-communicable diseases (SDG 1); promoting well-being for all at all ages (SDG 3); and boosting the consumption of algae and algae-based products from aquaculture as an alternative of fish consumption, contributing to restoring fish stocks and sustainable use of marine resources (SDG 14).

## Figures and Tables

**Table 1:** Fatty acids (FA) profile of green *Ulva rigida* and *Codium tomentosum*; red *Gracilaria* gracilis, *Palmaria palmata* and *Porphyra dioica*; and brown *Fucus vesiculosus* macroalgae. Abundances are expressed in relative abundance (%) and values are means of five samples (*n* = 5) ± standard deviation.

FA	*Ulva rigida*	*Codium tomentosum*	*Gracilaria gracilis*	*Palmaria palmata*	*Porphyra dioica*	*Fucus vesiculosus*
14:0		3.7 ± 0.4	3.2 ± 0.3	5.3 ± 0.4	9.3 ± 0.6	9.3 ± 0.5
16:0	20.2 ± 0.4	22.3 ± 1.2	27.1 ± 1.2	24.4 ± 1.1	23.3 ± 1.1	11.9 ± 0.5
16:1*n*-7	1.3 ± 0.1	4.9 ± 0.2	2.8 ± 0.8	2 ± 0.4	18.3 ± 0.7	1 ± 0.0
16:1*n*-9	2.1 ± 0.1	0.8 ± 0.0			0.9 ± 0.1	
16:2*n*-4		0.9 ± 0.1				
16:2*n*-6		0.8 ± 0.1			1.8 ± 0.1	
16:3*n*-4		1.7 ± 0.1			1.3 ± 0.1	
16:3*n*-6						1.4 ± 0.1
16:3*n*-3		10.3 ± 0.4				
16:4*n*-1		1.5 ± 0.1			3.5 ± 0.3	
16:4*n*-3	19 ± 0.6					
18:0	2.9 ± 1	2.6 ± 0.6	4.6 ± 0.8	12.5 ± 6.8	4.9 ± 1	3.6 ± 1.1
18:1 *	9.5 ± 0.3	11.1 ± 0.4	9.7 ± 0.4	2.8 ± 0.5	3.3 ± 0.2	22.2 ± 1.3
18:2*n*-6	1.5 ± 0.1	3.4 ± 0.1	2 ± 0.4		1.7 ± 0.1	8.5 ± 0.2
18:2*n*-3		3.6 ± 0.1				
18:3*n*-6	0.4 ± 0.1				2 ± 0.1	0.8 ± 0.0
18:3*n*-3	10.9 ± 0.4	14 ± 0.6	2.7 ± 0.2			6.7 ± 0.3
18:4*n*-3	24.4 ± 0.4	4.4 ± 0.1	7 ± 0.2		3.4 ± 0.2	6.2 ± 0.3
20:3*n*-6					2.4 ± 0.5	0.9 ± 0.1
20:4*n*-6		4.5 ± 0.4	35.4 ± 1.5	0.9 ± 0.2	2.7 ± 0.3	16.7 ± 0.7
20:4*n*-3	1.2 ± 0.1				0.6 ± 0.1	
20:5-*n*-3	1.4 ± 0.1	7.9 ± 0.8	5.5 ± 0.2	51.9 ± 6.5	20.5 ± 2.3	10.3 ± 0.5
22:0	1 ± 0.1	1.7 ± 0.4				0.3 ± 0.0
22:5*n*-3	4.1 ± 0.1					

* The 18:1 value is represented by the normalized sum of two peaks corresponding to C18:1 but whose unsaturation position was not clear to identify for all macroalgae.

**Table 2:** Fatty acids indicators of green *Ulva rigida* and *Codium tomentosum*; red *Gracilaria* *gracilis*, *Palmaria palmata* and *Porphyra dioica*; and brown *Fucus vesiculosus* macroalgae. Values correspond to relative abundances (except for AI and TI calculation) and are presented as average of five samples (*n* = 5) ± standard deviation. Different letters indicate statistically significant differences between macroalga species (*q* < 0.05, Kruskal–Wallis test followed by Dunn’s post-hoc comparisons).

Indicators	*Ulva rigida*	*Codium tomentosum*	*Gracilaria gracilis*	*Palmaria palmata*	*Porphyra dioica*	*Fucus vesiculosus*
SFA	24.1 ± 1.4	30.2 ± 1.6	34.9 ± 0.9	42.3 ± 7.3	37.5 ± 2.4	25.2 ± 2
MUFA	13 ± 0.3	16.8 ± 0.3	12.5 ± 0.7	4.9 ± 0.9	22.5 ± 0.7	23.3 ± 1.2
PUFA	62.9 ± 1.1	53 ± 1.4	52.6 ± 1.4	52.8 ± 6.7	40 ± 3	51.6 ± 1.5
PUFA omega-6	2 ± 0.1	8.7 ± 0.4	37.4 ± 1.3	0.9 ± 0.2	10.7 ± 0.8	28.3 ± 0.7
PUFA omega-3	60.9 ± 1.1	40.2 ± 1.3	15.1 ± 0.3	51.9 ± 6.5	24.5 ± 2.5	23.3 ± 0.9
AI	0.3 ± 0.0 ^a^	0.6 ± 0.1 ^a,b^	0.6 ± 0.0 ^a,c,d^	0.8 ± 0.1 ^c,e^	1.1 ± 0.1 ^e^	0.7 ± 0.1 ^b,d,e^
TI	0.1 ± 0.0 ^a^	0.2 ± 0.0 ^a,b^	0.5 ± 0.0 ^c^	0.2 ± 0.1 ^a,d^	0.4 ± 0.1 ^b,c^	0.3 ± 0.0 ^b,c,d^

SFA, saturated fatty acids; MUFA, monounsaturated fatty acids; PUFA, polyunsaturated fatty acids; AI, Atherogenicity index; TI, Thrombogenicity index. ^a^ There are no significant differences between *Ulva rigida*, *Codium tomentosum* and *Gracilaria gracilis*; ^b^ there are no significant differences between *Codium tomentosum* and *Fucus vesiculosus*; ^c^ there are no significant differences between *Gracilaria gracilis* and *Palmaria palmate*; ^d^ there are no significant differences between *Gracilaria gracilis* and *Fucus vesiculosus*; ^e^ there are no significant differences between *Palmaria palmata*, *Porphyra dioica* and *Fucus vesiculosus*.

**Table 3:** Lipid extract concentration (μg/mL) that provided inhibition of 50% (IC50) and 20% (IC20) for ABTS^●+^ and DPPH^●^ assays, respectively. Trolox equivalent (TE) [μmol of Trolox/g lipid] for radical scavenging activity. Values are presented as average of three assays (*n* = 3) ± standard deviation. Different letters in the same line represent significant differences among macroalgae (*q* < 0.05, Kruskal–Wallis test followed by Dunn’s post-hoc comparisons).

		*Ulva rigida*	*Codium tomentosum*	*Gracilaria gracilis*	*Palmaria palmata*	*Porphyra dioica*	*Fucus vesiculosus*
ABTS^●+^	IC50	30.7 ± 0.1 ^a,b^	48.1 ± 0.0 ^a,c^	86.4 ± 3.4 ^a^	23.7 ± 0.6 ^b,d^	41.1 ± 2.5 ^a,d^	27.3 ± 0.2 ^b,c,d^
	TE	500.5 ± 1.7 ^a,b^	327.9 ± 0.2 ^a,b^	183.0 ± 7.1 ^a^	606.1 ± 14.6 ^b^	338.8 ± 20.5 ^a,b^	507.1 ± 3.5 ^b^
DPPH^●^	IC20	120.8 ± 3.8 ^a,b^	249.9 ± 66.7 ^a^	119.5 ± 1.8 ^a,b^	119.6 ± 8.0 ^a,b^	212.5 ± 7.0 ^a^	106.0 ± 5.6 ^b^
	TE	88.0 ± 2.8	249.9 ± 66.7	89.2 ± 1.3	89.5 ± 6.3	44.9 ± 1.5	89.7 ± 4.6

**Table 4:** Polar lipid extract concentration (µg/mL] that induced 50% inhibition (IC50) of MDA-MB-231 cells. Values are means of three assays ± standard deviation.

Macroalgae	IC50
*Ulva rigida*	82.7 ± 19.1%
*Codium tomentosum*	66.4 ± 12.0%
*Gracilaria gracilis*	74.7 ± 19.1%
*Palmaria palmata*	40.4 ± 19.2%
*Pporphyra dioica*	35.5 ± 10.5%
*Fucus vesiculosus*	52.5 ± 10.9%
